# Supplementary material for: Expanding the Toolbox for Bicelle-Forming Surfactant–Lipid Mixtures
Source: Molecules. 2022 Nov 7;27(21):7628. doi: 10.3390/molecules27217628 (PMC9658636; doi:10.3390/molecules27217628)
Supplement: Supplementary file 1 [file molecules-27-07628-s001.zip › molecules-1995531-supplementary.pdf]

molecules

MDPI

Supporting Information

Table S1. Chemical composition, molecular volumes and electron densities of the lipids and detergent components. a is from [34], b is from [35], c was from [36] and d was estimated using the VegaZZ software [37].

Component	Chemical formula	Molecular volume (Å^3)	Electron density (e$^-$/ Å^3)
DMPC tails	$C_{26}H_{54}$	779.8^a	0.269
DMPC heads	$C_{10}H_{18}NO_8P$	320.9^a	0.489
DMPG heads	$C_8H_{12}O_{10}P$	283.0^b	0.494
POPC tails	$C_{32}H_{64}$	937.2^a	0.273
DHPC tails	$C_{10}H_{22}$	330.2^a	0.248
CHAPS	$C_{32}H_{58}N_2O_7S$	830.3^c	0.405
Cholate	$C_{24}H_{39}O_5$	473.0^d	0.471

Table S2. Complete list of the values describing the fits obtained for the curves shown in Figure 3, Figure 5 and Figure S2 and plotted in Figure 4B, Figure 5B and Figure S7.

q	Scale	BKG	Radius	X_core	Thick_rim	Thick_face	Length	SLD_core	SLD_face	SLD_rim	SLD_solvent	Belt_rough	ChiSq
DMPC_CHAPS													
0.5	1.25E-02	6.28E-04	19.6	0.705	10.7	6.4	19.8	7.6	11.85	11.18	9.27	3	1.27
1	8.69E-03	4.32E-04	26.0	0.667	8.8	9.5	24.1	7.6	11.74	11.29	9.27	3	1.34
1.5	7.35E-03	2.61E-04	31.4	0.685	8.0	10.3	23.3	7.6	11.80	11.25	9.27	3	1.45
2	6.73E-03	2.67E-04	34.5	0.675	7.8	10.5	23.8	7.6	11.83	11.23	9.27	3	1.65
2.5	6.34E-03	2.61E-04	34.8	0.672	8.4	9.7	24.5	7.6	11.89	11.27	9.27	3	1.90
3	6.13E-03	2.60E-04	37.3	0.654	8.3	10.0	24.7	7.6	11.84	11.26	9.27	3	1.73
5	5.67E-03	2.16E-04	39.0	0.688	8.6	9.9	25.0	7.6	11.88	11.23	9.27	3	1.82
DMPC_Chol													
0.5	8.89E-03	1.00E-03	12.4	0.884	12.2	4.0	14.9	7.6	11.00	11.27	9.27	3	1.46
1	7.69E-03	7.00E-04	15.8	0.832	10.4	4.5	20.8	7.6	11.17	11.43	9.27	3	1.46
1.5	6.33E-03	7.00E-04	22.2	0.920	4.2	11.5	17.6	7.6	11.69	11.42	9.27	3	1.46
2	5.92E-03	7.50E-04	23.0	0.877	4.4	11.5	18.2	7.6	11.72	11.40	9.27	3	1.38
2.5	5.83E-03	6.00E-04	24.0	0.919	4.3	10.8	18.9	7.6	11.97	11.22	9.27	3	1.37
3	5.38E-03	6.80E-04	25.6	0.843	4.0	10.3	19.9	7.6	12.09	11.54	9.27	3	1.36
5	5.38E-03	6.80E-04	25.6	0.843	4.0	10.3	19.9	7.6	12.09	11.54	9.27	3	1.36
DMPC_DHPC													
0.5	1.07E-02	5.45E-04	30.1	0.694	4.5	8.3	24.4	7.6	11.63	11.97	9.27	3	1.52
1	8.02E-03	3.96E-04	41.2	0.738	4.3	9.6	24.2	7.6	11.60	11.78	9.27	3	1.43
1.5	6.71E-03	2.14E-04	45.6	0.728	4.3	9.4	25.3	7.6	11.81	11.81	9.27	3	1.31
2	6.45E-03	2.00E-04	46.5	0.769	4.3	9.7	25.6	7.6	11.83	11.78	9.27	3	1.51
2.5	6.06E-03	2.66E-04	46.6	0.802	4.1	10.0	25.7	7.6	11.77	11.81	9.27	3	1.70
3	5.89E-03	2.16E-04	45.1	0.888	4.1	10.1	25.7	7.6	11.83	11.59	9.27	3	1.64
5	5.56E-03	2.68E-04	50.1	0.830	4.1	10.5	25.6	7.6	11.78	11.48	9.27	3	1.92
DMPC_PG_CHAPS													
0.5	1.07E-02	8.85E-04	20.2	0.627	10.9	7.4	22.0	7.6	11.79	11.28	9.27	3	2.15
1	7.80E-03	5.11E-04	27.8	0.635	9.1	10.0	23.3	7.6	11.86	11.26	9.27	3	1.65
1.5	6.80E-03	3.06E-04	31.6	0.684	8.5	10.7	23.6	7.6	11.80	11.27	9.27	3	1.55
2	6.30E-03	3.02E-04	35.1	0.670	8.5	10.8	23.8	7.6	11.80	11.23	9.27	3	1.33
2.5	6.00E-03	3.34E-04	37.1	0.700	8.0	10.5	24.2	7.6	11.98	11.21	9.27	3	1.40
3	5.80E-03	3.00E-04	39.8	0.667	8.1	11.0	24.0	7.6	11.83	11.24	9.27	3	2.00
5	5.50E-03	2.17E-04	40.9	0.725	9.6	11.1	24.1	7.6	11.74	11.04	9.27	3	1.68
DMPC_PG_Chol													
0.5	9.15E-03	1.00E-03	13.1	0.938	11.5	4.1	15.8	7.6	11.50	11.33	9.27	3	2.48
1	7.58E-03	9.38E-04	17.6	0.661	10.5	4.1	24.8	7.6	11.82	11.32	9.27	3	2.38
1.5	6.11E-03	7.50E-04	23.4	0.869	4.1	11.6	18.0	7.6	11.85	11.39	9.27	3	1.44
2	6.08E-03	7.10E-04	24.7	0.869	4.1	11.6	18.3	7.6	11.88	11.25	9.27	3	1.46
2.5	5.75E-03	9.22E-04	26.1	0.847	4.7	10.9	19.5	7.6	12.12	11.03	9.27	3	1.78
3	5.70E-03	7.14E-04	25.0	0.880	4.7	10.3	19.9	7.6	12.26	11.15	9.27	3	1.42
5	5.55E-03	7.00E-04	24.3	0.976	4.5	10.8	19.6	7.6	12.08	11.16	9.27	3	1.53
DMPC_PG_DHPC													
0.5	1.08E-02	6.78E-04	32.4	0.694	4.3	9.4	23.4	7.6	11.57	11.85	9.27	3	1.25
1	7.67E-03	4.41E-04	45.6	0.630	4.3	9.7	24.7	7.6	11.69	11.86	9.27	3	1.43
1.5	6.78E-03	2.17E-04	47.2	0.661	4.9	9.9	25.7	7.6	11.69	11.87	9.27	3	2.18
2	6.21E-03	2.60E-04	47.5	0.690	5.1	10.2	25.8	7.6	11.67	11.82	9.27	3	1.30
2.5	6.07E-03	3.17E-04	51.7	0.785	4.2	10.8	25.2	7.6	11.72	11.72	9.27	3	1.45
3	6.27E-03	2.66E-04	48.2	0.938	4.2	11.0	24.9	7.6	11.68	11.69	9.27	3	1.33
5	5.58E-03	2.61E-04	55.3	0.885	4.1	11.9	24.9	7.6	11.64	11.33	9.27	3	1.41

POPC_DHPC													
0.5	2.07E-02	3.57E-04	28.1	0.731	6.6	5.6	29.6	7.7	12.96	11.03	9.27	3	3.04
1	1.68E-02	1.43E-04	38.0	0.573	6.5	5.4	29.9	7.7	12.57	11.45	9.27	3	2.88
1.5	1.06E-02	1.28E-04	137.3	0.197	7.7	11.4	24.6	7.7	11.00	11.00	9.27	3	1.45
2	9.94E-03	1.20E-04	198.9	0.136	7.6	11.5	24.6	7.7	11.00	11.02	9.27	3	1.39
2.5	7.39E-03	1.12E-04	245.7	0.124	6.1	11.3	24.2	7.7	11.00	11.61	9.27	3	1.52
3	6.47E-03	1.13E-04	247.8	0.124	7.8	11.6	24.2	7.7	11.00	11.01	9.27	3	1.68
5	4.47E-03	8.38E-05	943.0	0.033	4.8	10.7	24.6	7.7	11.13	12.59	9.27	3	2.38

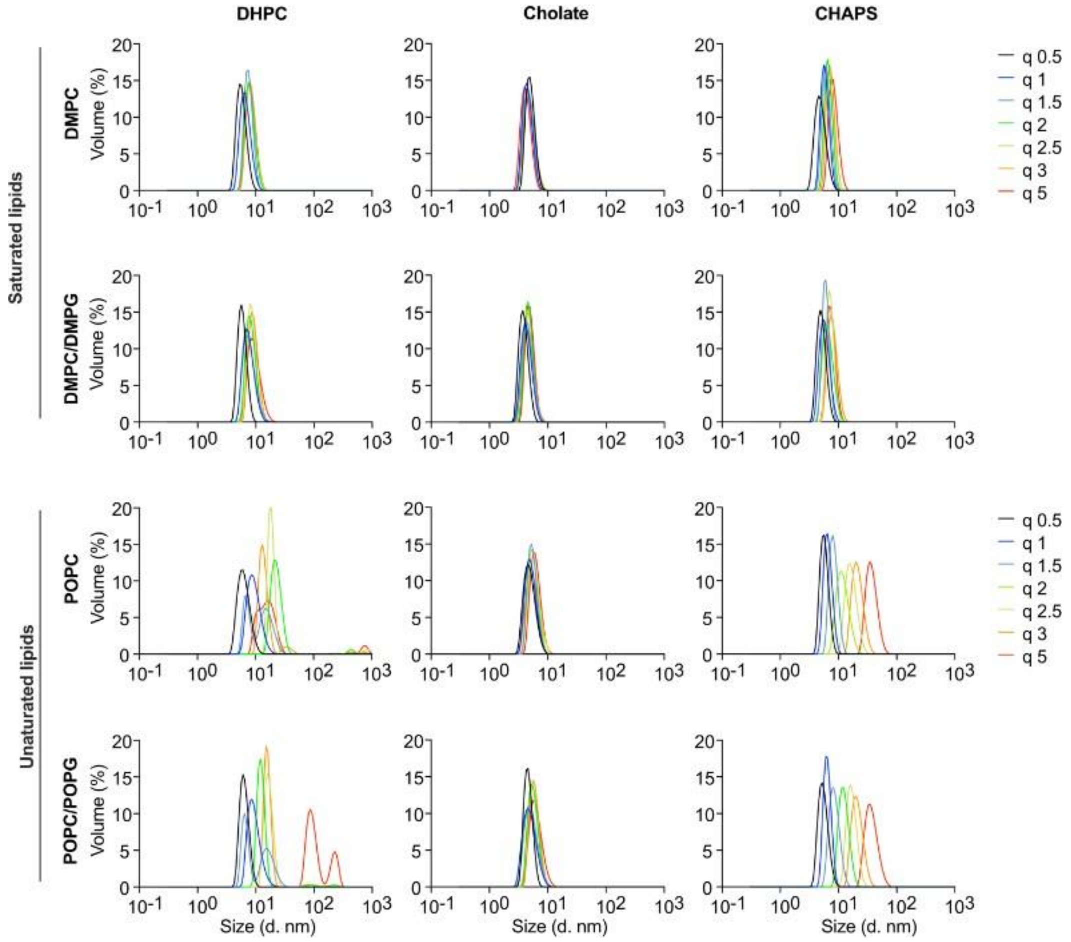

Figure S1. DLS size distributions of the lipid-detergent mixtures measured in this study. The curves are representative of one to three independent experiments.

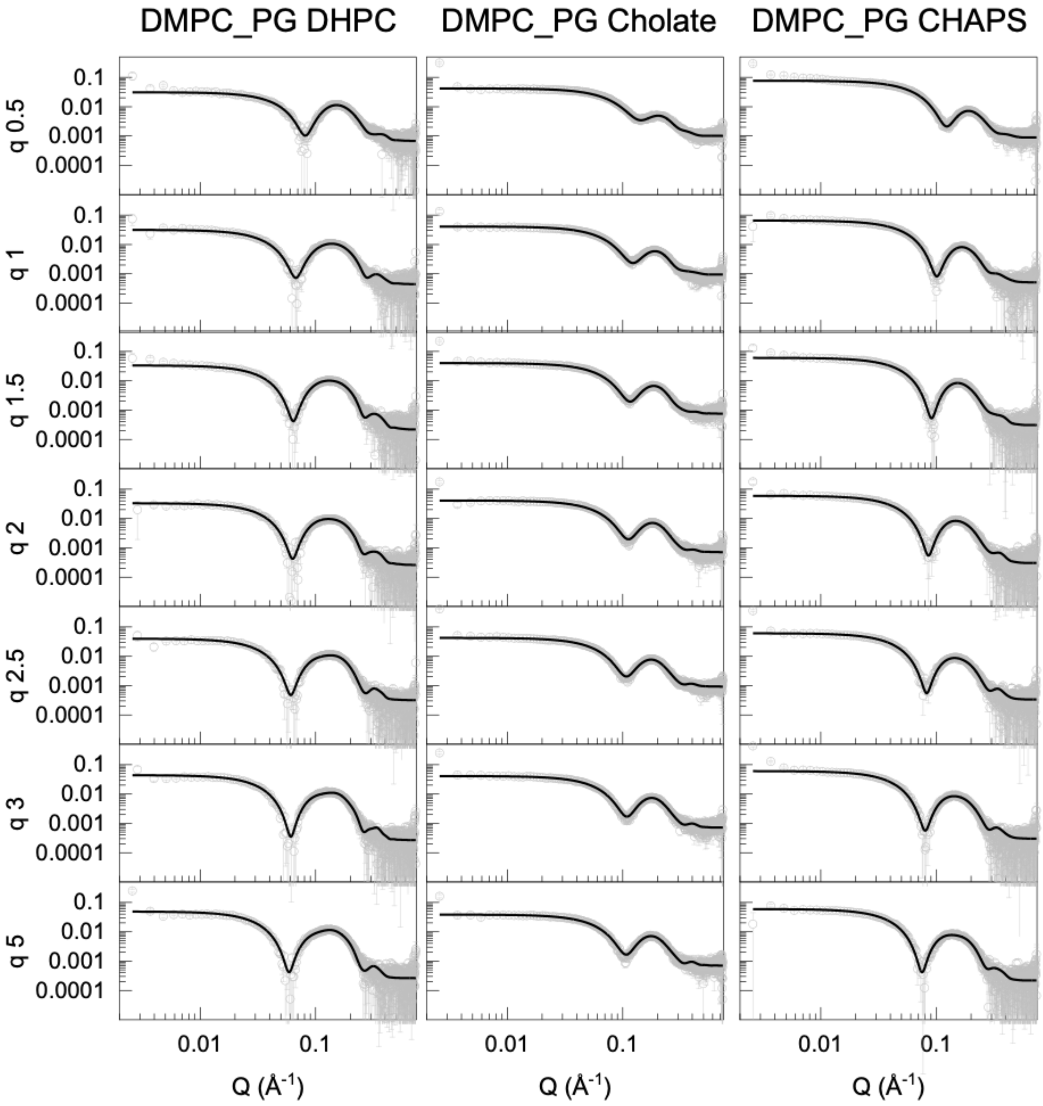

Figure S2. SAXS curves and best fit to the data for the systems containing DMPC-DMPG (80:20) and the three detergents.

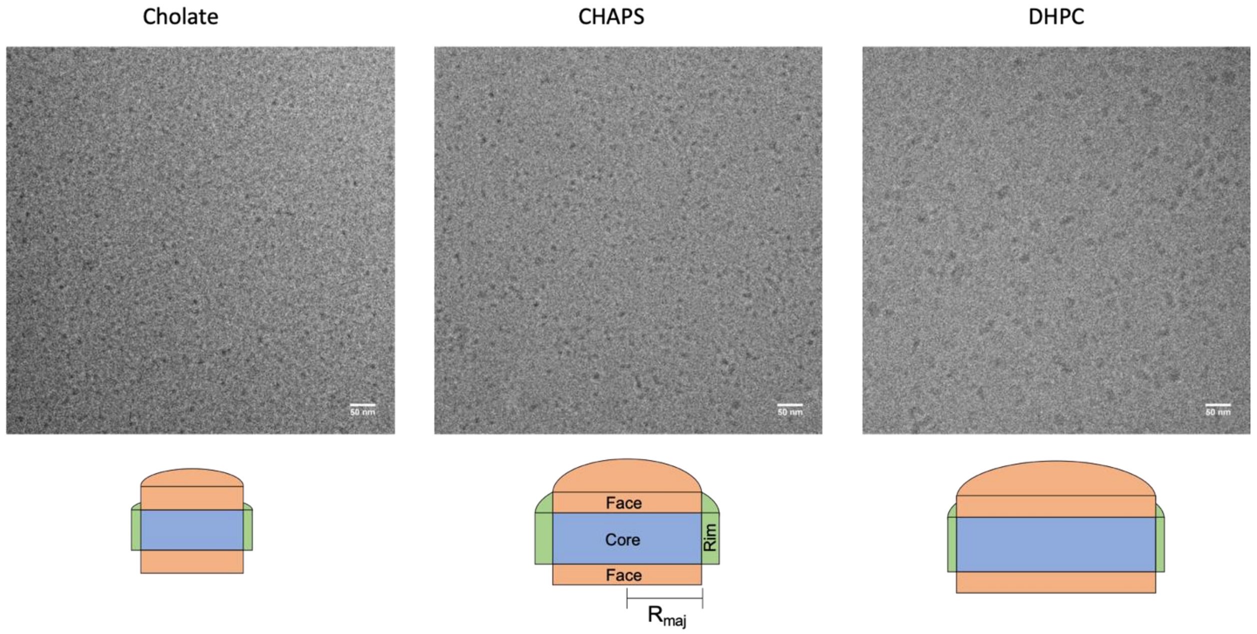

Figure S3. Cryo EM images of DMPC mixed with cholate (left) CHAPS (centre) and DHPC (left) at a q ratio of 2.5, scale bar = 50 nm. The schematic cartoons underneath represent the structures obtained from the fitting of the SAXS data drawn to scale using the thickness parameters obtained from the fits.

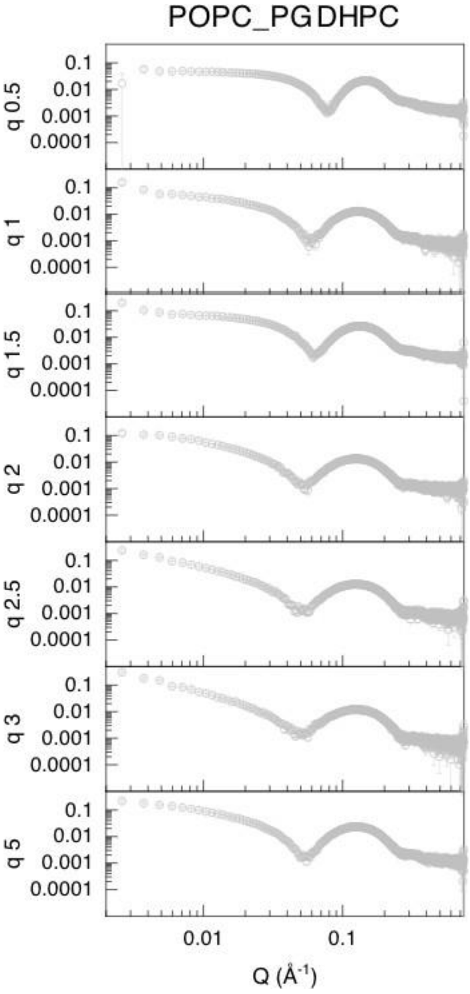

Figure S4. SAXS curves for the POPC-POPG (80:20) samples mixed with DHPC.

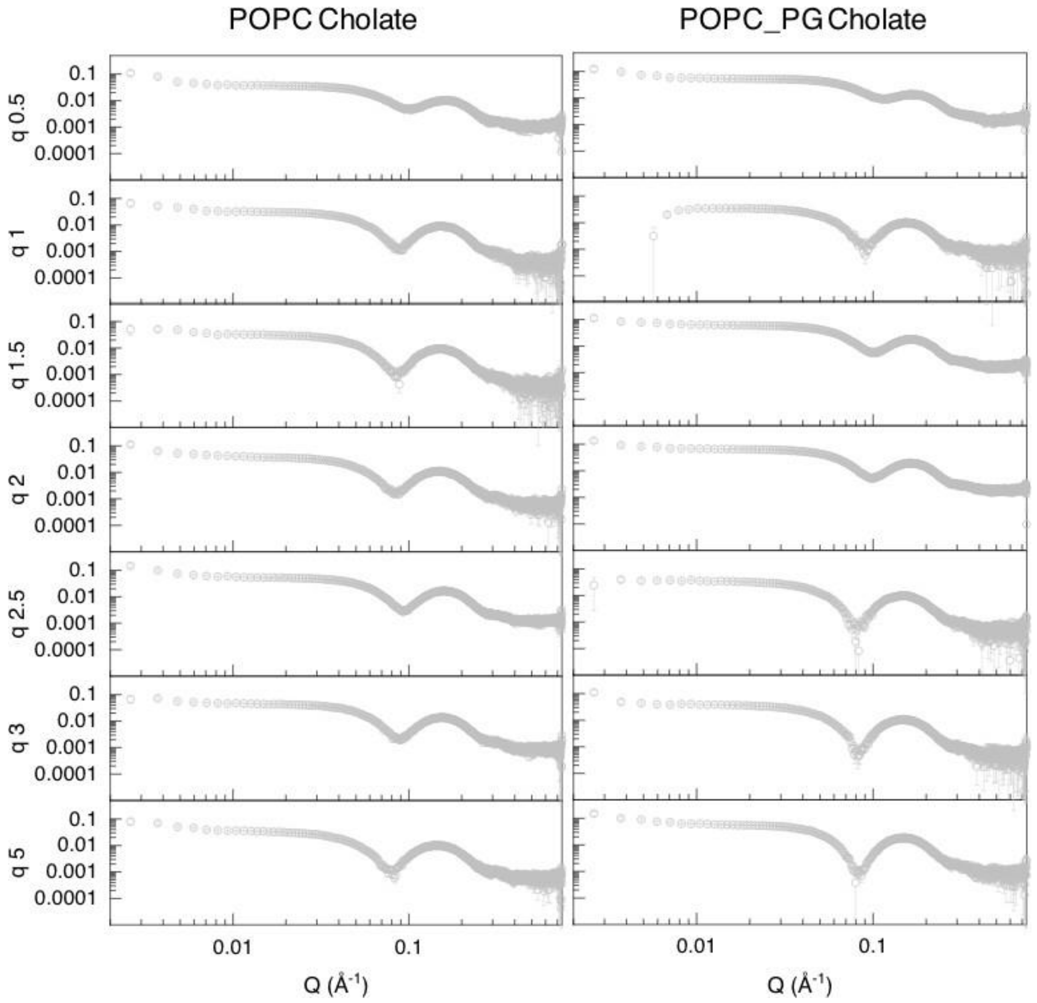

Figure S5. SAXS curves for the POPC and POPC-POPG (80:20) samples mixed with sodium cholate.

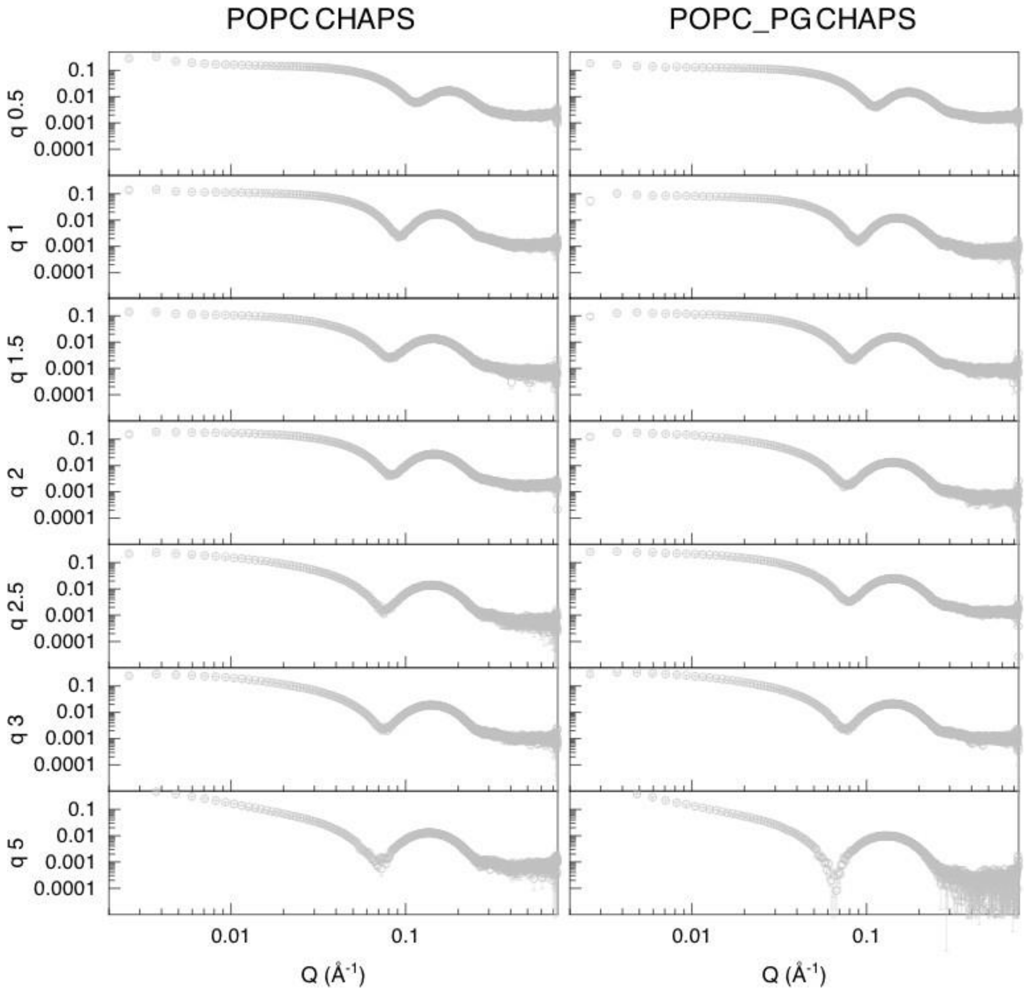

Figure S6. SAXS curves for the POPC and POPC-POPG (80:20) samples mixed with CHAPS.

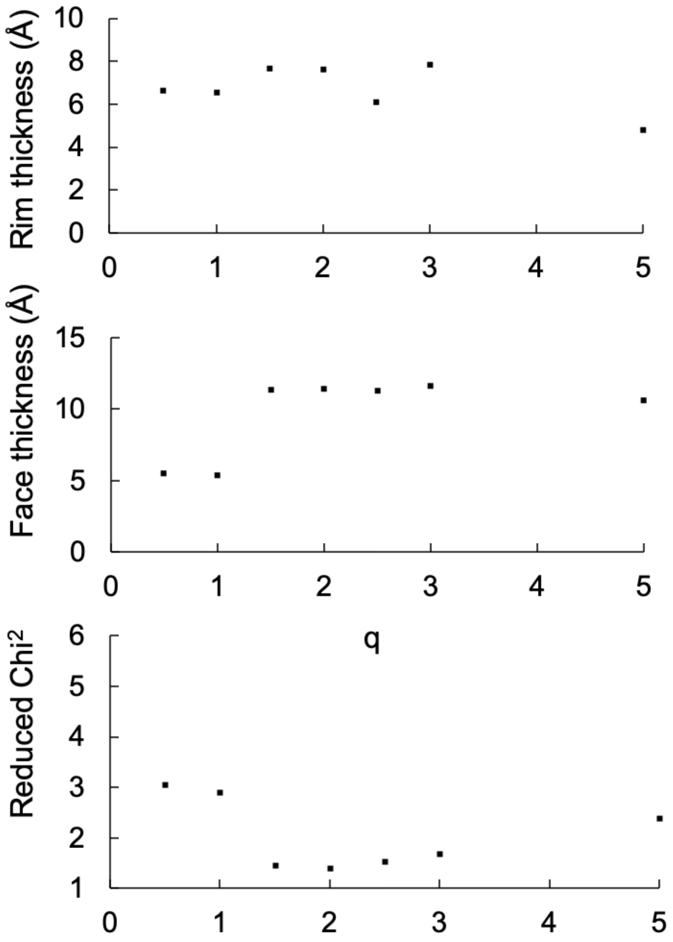

Figure S7. Remaining fit parameters for the POPC-DHPC fits.

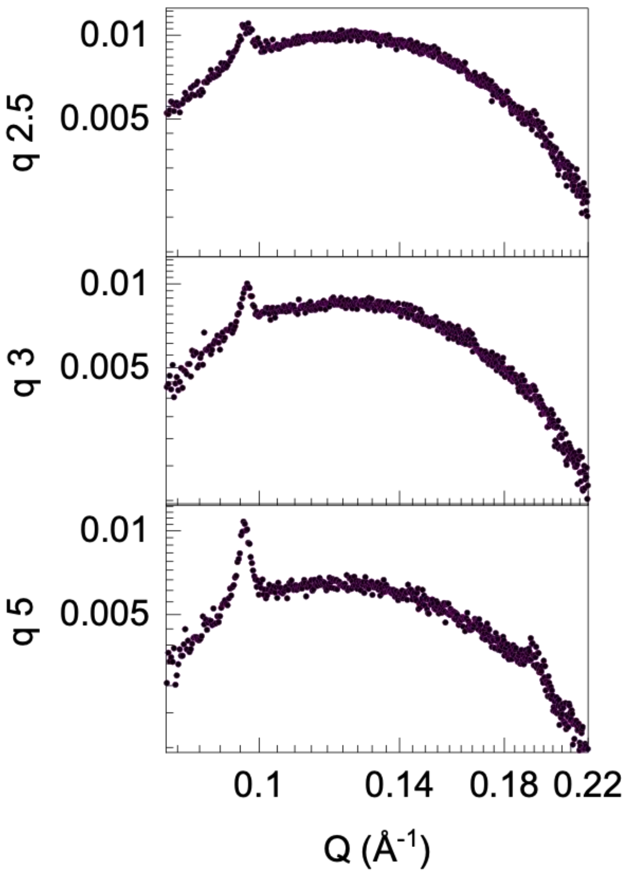

Figure S8. Evolution of the Bragg peaks in the POPC-DHPC sample at q = 2.5, q = 3 and q=5.
